# Heavy Metal Accumulation and the Genotoxicity in Barbel (*Barbus barbus*) as Indicators of the Danube River Pollution

**DOI:** 10.1100/2012/351074

**Published:** 2012-04-26

**Authors:** Karolina Sunjog, Zoran Gačić, Stoimir Kolarević, Željka Višnjić-Jeftić, Ivan Jarić, Jelena Knežević-Vukčević, Branka Vuković-Gačić, Mirjana Lenhardt

**Affiliations:** ^1^Institute for Multidisciplinary Research, University of Belgrade, 11000 Belgrade, Serbia; ^2^Faculty of Biology, University of Belgrade, 11000 Belgrade, Serbia; ^3^Institute of Biological Research, University of Belgrade, 11000 Belgrade, Serbia

## Abstract

The aim of this study was to analyze 16 trace elements (Al, As, B, Ba, Cd, Co, Cr, Cu, Fe, Li, Mn, Mo, Ni, Pb, Sr, and Zn) in different barbel (*Barbus barbus*) tissues and to detect the presence of genotoxic effects in erythrocytes with the alkaline comet assay. Barbel specimens were collected in the Danube river near Belgrade, Serbia, where the discharge of untreated communal and industrial wastewaters is likely to produce negative effects on fish residing in this area. The highest concentrations of Sr, Mn, Fe, Ba, B, and Al were found in gills, Mo and Cu in liver, and As and Zn in gonads. Concentrations of Zn and Fe were above maximum acceptable concentrations (MACs) in a number of gonad, gill, and liver samples. Three-year-old barbel specimens had higher tail moment and Zn concentrations in gills (1.71 and 51.20 **μ**g/g dw, resp.) than 5-year-old specimens (0.85 and 42.51 **μ**g/g dw, resp.). Results indicate that the younger barbel specimens might be more suitable for the monitoring of environmental pollution.

## 1. Introduction

Intensive industrialization and different anthropogenic influences have resulted in an increased presence of contaminants in aquatic habitats, thus requiring permanent monitoring of the presence of pollutants and their impact [1]. The Danube river in Serbia receives untreated wastewaters from a number of industrial facilities that are situated along the river [2, 3], especially in the vicinity of large cities [4]. Milijašević et al. [5] estimated that approximately 90% of all industrial wastewaters in Serbia are being discharged into water bodies without previous treatment. Furthermore, sediments in the Danube and its tributaries, such as the Tisza and the Sava rivers, contain increased levels of Zn, Mn, and Pb [6]. Chemical analyses of toxicant concentrations in the environment or in biological samples are not sufficient if their impact on the aquatic biota is not also properly evaluated [7, 8].

 Aquatic ecosystems receive a number of toxic substances, among which heavy metals are of significant importance, due to their toxicity, their bioaccumulation potential, and their ability to induce damage in DNA [9]. Fish are often used as sentinel organisms due to their role in food webs, their potential for bioaccumulation of toxic substances, and their sensitivity to even low concentrations of mutagens. Investigations conducted on sterlet (*Acipenser ruthenus*) and the Pontic shad (*Alosa immaculata*) from the Serbian part of the Danube River have revealed that concentrations of Cd, Pb, and Fe had exceeded maximum acceptable concentration (MAC) prescribed by both EU and national legislation [10, 11].

It is well known that a number of chemicals have a high persistency when they are released into an environment and have mutagenic and/or clastogenic properties [12]. The analysis of DNA alterations in aquatic organisms is widely accepted as a suitable method for evaluating genotoxic contamination of the environment and can be used to detect exposure in a wide range of species [12]. The comet assay, or a single cell gel electrophoresis, has a wide application as a simple and sensitive method for evaluating DNA damage in fish exposed to various xenobiotics in the aquatic environment [12, 13]. The alkaline comet assay was approved in fish as more sensitive to the genotoxicity of river contaminants than the micronucleus test [14]. Unlike classical chromosome breakage studies, such as micronucleus formation or cell survival studies, comet assay allows scanning complete genomes in all cell cycles, rather than just during mitosis.

The aim of the present study was to acquire information on concentrations of heavy metals in gills, muscle, liver, and gonads of barbel (*Barbus barbus*) from the Serbian part of the Danube river and on the possible biological effects of the pollution on this species, through the analysis of DNA damage.

## 2. Materials and Methods

### 2.1. Sample Collection and Preparation

Barbel is a commercially important fish species in the Serbian part of the Danube river. As a bottom feeder, it is exposed to toxicants from both water and sediments. A total of 10 specimens of barbel were collected at the end of March 2010 by professional fishermen from the Danube (river kilometer 1173) in the vicinity of Belgrade, in the area where the river receives untreated communal and wastewaters from the city. Fish specimens were anesthetized with clove oil, and the blood was immediately collected from the heart with heparinized syringes. The collected blood for comet assay was stored on ice and transferred to the laboratory in a dark cool box. The total mass (g) and the total body length (cm) of each specimen were measured. Samples of liver, gills, muscle, and gonads were quickly removed, washed with distilled water, and stored at −20°C prior to analysis. Age determination was performed analyzing the growth marks of scales. It was not possible to determine the sex by macroscopic inspection of gonads. Comparison for DNA damage was made with 4 specimens caught from an unpolluted reference site (Uvac reservoir −43°25'N, 19°55'E).

### 2.2. Heavy Metal and Trace Elements Analysis

Samples were dried by Freeze Dryers Rotational Vacuum Concentrator, GAMMA 1–16 LSC, Germany, and sample portions between 0.2 and 0.5 g (dry weight) were processed in a microwave digester (speed wave MWS-3+; Bergof Products+ Instruments GmbH, Eningen, Germany), using 6 mL of 65% HNO_3_ and 4 mL of 30% H_2_O_2_ (Merck suprapure) at a food temperature program (100–170°C). The digested samples were diluted with distilled water to a total volume of 25 mL, and the analysis was performed by inductively coupled plasma optical spectrometry (ICP-OES). The following 16 heavy metals and trace elements were analyzed: Al, As, B, Ba, Cd, Co, Cr, Cu, Fe, Li, Mn, Mo, Ni, Pb, Sr, and Zn. The following wavelength lines of the ICP-OES analysis were used: Al 394.401, As 189.042, B 249.773, Ba 233.527, Cd 228.802, Co 228.616, Cr 205.552, Cu 324.754, Fe 259.941, Li 460.289, Mn 259.373, Mo 202.095, Ni 231.604, Pb 220.353, Sr 460.733, and Zn 206.191. BCR-185R reference material of bovine liver as well as IAEA-336 Lichen reference material was used for the assessment of the accuracy and precision of the analysis. Analysis indicated that the concentrations were within 90–115% of the certified values for all measured elements. All heavy metal concentrations were expressed as *μ*g g^−1^ dry weight (dw). 

### 2.3. Comet Assay

The alkaline comet assay procedure was performed under yellow light, in accordance with the method described by Singh et al. [15]. Microscopic slides were precoated with 0.5% NMP agarose and air dried for 24 h. To form the second, supportive layer, 80 *μ*L of 1% NMP agarose, was gently placed on the top of the 0.5% NMP layer and spread over the slide using coverslip. The slide was placed on ice for 5 min to allow complete polymerization of the agarose. After the coverslips were removed, 30 *μ*L of erythrocyte pellet suspension, gently mixed with 70 *μ*L of 1% LMP (37°C) agarose, was pipetted on the supportive layer of 1% NMP agarose and covered with a coverslip. After keeping coverslips for 5 min on ice, they were removed and the slides were placed into freshly made cold lysis buffer (2.5 M NaCl, 100 mM EDTA, 10 mMTris, 1.5% Triton X-100, 10% Dimethyl sulfoxide, pH 10) for 1 hour. To allow DNA unwinding, slides were put into electrophoresis chamber containing cold alkaline electrophoresis buffer (300 mM NaOH, 1 mM EDTA, pH 13) for 20 min. Electrophoresis was performed at 0.75 V/cm at 4°C for 20 min. After the electrophoresis, slides were placed into freshly made neutralizing buffer (0.4 M Tris, pH 7.5) for 15 min. Staining was performed with 20 *μ*L per slide of EtBr (2 *μ*g/mL). The slides were examined with a fluorescence microscope (Leica, DMLS, Austria, 400x magnification, 510–560 nm excitation filter, 590 nm barrier filter). Microscopic images of comets were scored using Comet IV Computer Software (Perceptive Instruments, UK). Images of 25 cells were collected from each of the two replicate slides per sample, and, among the parameters available for analyses, the tail moment, tail length, and tail intensity were used to assess DNA damage.

### 2.4. Statistical Analysis

All statistical analyses of data were performed by Statistica for Windows [16]. Canonical Discriminant Analysis (CDA) was utilized to evaluate differences among tissues in heavy metal accumulation. Mann-Whitney *U*-test was used to compare concentrations of 10 heavy metals in 4 analyzed tissues, as well as differences between sites and between fish of different age.

## 3. Results and Discussion

The weight and the total body length of the studied barbel ranged from 205 to 671 g and from 29 to 41 cm, respectively. According to the age assessment, specimens were between 3^+^ (in the fourth year of life) and 5^+^ (in the sixth year of life) old. Heavy metal and trace element concentrations in each of the four analyzed tissues are presented in Table 1. Concentrations of Cd, Co, Cr, Li, Ni, and Pb were below the detection threshold level in all samples.

Comparisons of the extent of accumulation among analyzed tissues showed that the differences were statistically significant for most of the assessed metals and trace elements (*P* < 0.05, Table 1).

 Concentrations of Sr, Mn, Fe, Ba, B, and Al were highest in gills, which made gills the most affected tissue. Such results are consistent with the literature. In fish, gills represent the largest surface in contact with the aquatic environment and they are thus expected to show the greatest tissue alteration, genotoxicity, and the accumulation of toxic chemicals [17]. As a consequence, respiratory function of gills can become decreased, consequently affecting the general health and ultimately resulting in death of the affected individual [18].

Concentrations of Mo and Cu were highest in the liver, while As and Zn had the highest concentrations in gonads. Studies of Bagdonas and Vosyliene [19], and Obiakor et al. [20] revealed that even a short-term exposure of fish to Cu and Zn in concentrations higher than those prescribed as MAC could produce genotoxic effects on erythrocytes. These studies also emphasized synergistic effects of these two metals.

**Figure 1:**
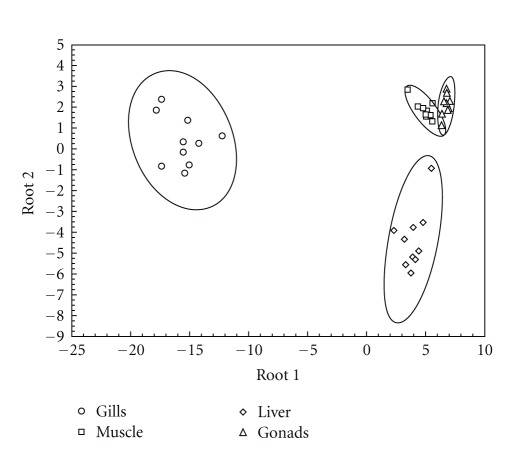
Results of the Canonical Discriminant Analysis applied on the four studied tissues (ellipses show 90% confidence intervals). The input variables are heavy metal and trace element concentrations in each barbel tissue.

CDA showed a clear differentiation among the four analyzed barbel tissues (Figure 1). The first two canonical functions accounted for 77.04% of the total heterogeneity (Root1 −55.83% and Root2 −21.21%). Gills were separated from the other three tissues along the first canonical function, while the liver was mostly differentiated from other tissues along the second canonical function. Gonads were differentiated by the third canonical function (Root3 −17.79%). Liver was mostly differentiated by high concentrations of Mo, gonads by high concentrations of As, and gills by high concentrations of Sr. 

Comparison of heavy metals and trace elements between 3^+^- and 5^+^-year-old barbel specimens showed only significant difference in concentrations of Zn in gills, with higher values present in 3^+^-year-old fish (51.20 ± 4.25 *μ*g/g dw) than in 5^+^-year-old fish (42.51 ± 2.82 *μ*g/g dw). Thielen et al. [21] and Nachev et al. [1] found higher concentrations of heavy metals and trace elements in helminths than in barbel, their host, from the Hungarian and Bulgarian part of the Danube River. These authors also found negative correlation between the number of parasites and the level of metal accumulation in organs of the barbel. Our investigation revealed a higher occurrence of the parasite *Pomphorhynchus laevis *(Acanthocephala) in the older barbel specimens than in the younger ones (128 ± 7.8 and 72.5 ± 21.5, resp.). Significantly higher concentrations of Zn in gills in younger barbel specimens, as well as a lower number of parasites, indicate potential existence of the negative correlation between the abundance of parasites and the metal levels in barbel organs. Linde et al. [22] found that the older trouts (*Salmo trutta*) seem less appropriate for the monitoring of heavy metal pollution than the younger specimens. Results obtained in the present study indicate that the younger barbels seem to be more suited for the monitoring of environmental pollution, due to their higher susceptibility. 

In some tissues, concentrations of heavy metals exceeded acceptable levels for human consumption. Concentrations of Zn were above limits recommended by FAO (30 *μ*g/g wet weight) in five gonad samples. Concentrations of Fe were above the national MAC (30 *μ*g/g wet weight) in five gill and two liver samples. 

The three parameters of genotoxicity—tail moment, tail length, and tail intensity—have been widely used by researchers for evaluating DNA damage. As the amount of damage increases in a cell, more DNA migrates into the tail region and is quantified in terms of an increased amount of determined fluorescence in the tail region, as well as by tail length. The ratio of the DNA in the tail region (tail intensity) is commonly used for quantifying DNA strand breakage and represents the most reliable parameter [23]. However, the main disadvantage of this parameter is that two cells with different tail lengths may have the same degree of tail intensity and may produce the same result. A major advantage when using the tail moment as an index of the DNA damage is that both the amount of the damaged DNA and the distance of the genetic material migration in the tail are represented by a single number [24]. Which parameter of DNA migration will be applied often depends on the personal preferences of the investigator. In this study, we used all three parameters and conducted the scoring with the comet assay IV software, to reduce the possibility of bias due to subjectivity. 

The results of the comet assay analysis are summarized in Table 2. We compared the comet parameters of the fish from the Danube river with those from Uvac reservoir, Special Nature Reserve, as a control site with a very low anthropogenic influence. We observed differences between the three- and five-year fish, with significant differences in two of the three assessed parameters (tail moment and tail intensity), which is in accordance with previous studies [25]. All three parameters showed significant differences when the studied site and the reference site were compared. The tail intensity in fish from the Danube river (7.92) was significantly higher (*P* < 0.001) than in fish from the reference site (4.76). Furthermore, the tail moment and the tail length parameters in fish from the Danube river (1.28 and 28.51, resp.) were both significantly higher (*P* = 0) than in fish from the reference site (0.31 and 12.80, resp.). Tail moment in carp (*Cyprinus carpio*) from the Lake Mogan, which is under pollution stress of agricultural activities, was 1.50 ± 1.48 [24], which is a similar result to the one obtained in the present study (1.28 ± 1.41). 

Fish can serve as useful genetic models for the evaluation of pollution in aquatic ecosystems [26]. Marcon et al. [27] found that a high content of certain metals in the water could be one of the factors that might cause genetic damages in fish. The comet assay has been successfully used to investigate effects of genotoxic pollutants on the integrity of DNA [24, 28–30]. There are numerous studies dealing with the comet assay conducted *in vitro* [29–33], while few studies have been focused on *in situ* genotoxicity [34]. Micronucleus test was the only genotoxicity test applied thus far on barbel specimens from the natural population in the Danube river [33]. Therefore, the present study represents the first application of the comet assay for the evaluation of the Danube river water genotoxicity on barbel. 

Our results confirm earlier claims that fish represent a good model system for water monitoring. Biomarkers of genotoxicity have confirmed their sensitivity and suitability for this type of research. Since heavy metals do not represent the only group of water pollutants, it is very likely that other types of pollutants have also contributed to the increase in genotoxicity, determined in the present study. White and Rasmussen [35] presented evidence that despite the substantial genotoxicity of some industrial wastewaters, domestic wastewaters constitute a major genotoxic hazard to aquatic ecosystems. Since the industrial and domestic wastewaters in Serbia are still not processed before being released into watercourses, pollution indicators and genotoxicity parameters represent an essential tool for efficient monitoring of aquatic ecosystems in Serbia.

## Figures and Tables

**Table 1:** Heavy metal and trace element concentrations in different tissue of barbel (*Barbus barbus*) (mean value ± standard deviation). Concentrations are expressed as *μ*g/g dry weight, while ND indicates the values below the detection threshold.

		Muscle	Liver	Gills	Gonads
Al	Mean ± SD	ND	4.88, 92.76**	44.23 ± 71.08	ND
	min–max			4.80–243.38	
As	Mean ± SD	1.40*	0.54*	0.59*	1.41 ± 0.90
	min–max				0.18–2.71
B	Mean ± SD	ND	ND	10.71 ± 5.70	ND
	min–max			5.95–23.72	
Ba	Mean ± SD	ND	ND	10.63 ± 7.07	ND
	min–max			4.26–24.73	
Cu	Mean ± SD	ND	27.49 ± 27.05	ND	ND
	min–max		9.32–58.58		
Fe	Mean ± SD	ND	78.82 ± 24.66^a^	120.91 ± 32.91^b^	ND
	min–max		24.07–121.22	78.68–161.50	
Mn	Mean ± SD	ND	0.78 ± 0.64^a^	7.76 ± 2.54^b^	ND
	min–max		0.27–1.64	4.53–12.23	
Mo	Mean ± SD	ND	0.27 ± 0.11	0.06, 0.81**	ND
	min–max		0.11–0.40		
Sr	Mean ± SD	4.38 ± 2.24^a^	0.11 ± 0.07^b^	59.60 ± 12.93^c^	0.07 ± 0.01^d^
	min–max	2.04–9.77	0.06–0.28	36.63–77.03	0.06–0.08
Zn	Mean ± SD	12.89 ± 11.72^a^	47.08 ± 18.44^b^	47.85 ± 5.53^b^	71.69 ± 53.94^b^
	min–max	4.72–29.62	21.57–85.06	39.86–57.68	5.21–171.89

^a,b,c,d^The value with a different letter in the same row is different (Man-Whitney *U*-test, *P* < 0.05).

*Concentrations above detection threshold only in one sample.

**Concentrations above detection threshold only in two samples.

**Table 2:** Results of the comet assay applied on barbel (*Barbus barbus*) specimens from the Danube river and the reference site.

Location	Number of cells	Age	Tail length (*μ*m)	Tail moment	Tail intensity (%)
River Danube, Zemun	150	3^+^	28.35 ± 10.93	1.71 ± 1.65	10.77 ± 10.93
	150	5^+^	28.67 ± 7.80	0.86 ± 1.17	5.07 ± 7.01
	Total 300		28.51 ± 9.36	1.28 ± 1.41	7.92 ± 8.97
Reference site, Uvac	200		12.80 ± 4.31	0.31 ± 0.37	4.76 ± 5.36
